# Three Draft Genome Sequences of the Bacterial Plant Pathogen* Ralstonia solanacearum*, Isolated in Georgia

**DOI:** 10.1128/genomeA.00480-17

**Published:** 2017-06-08

**Authors:** Adam Kotorashvili, Galina Meparishvili, Giorgi Gogoladze, Nato Kotaria, Maka Muradashvili, Maia Zarandia, Daviti Tsaguria

**Affiliations:** a Lugar Center for Public Health Research at National Center for Disease Control and Public Health, Tbilisi, Georgia; b Institute of Phytopathology and Biodiversity of Batumi State University, Batumi, Georgia

## Abstract

*Ralstonia solanacearum*, the causative agent of bacterial wilt, is a devastating bacterial plant pathogen with a wide range of hosts. We report here the first draft genome sequences for three strains of *Ralstonia solanacearum* isolated from infected potato, tomato, and pepper plants in Georgia.

## GENOME ANNOUNCEMENT

*Ralstonia solanacearum* is an aerobic non-spore-forming, Gram-negative, plant-pathogenic bacterium that can cause bacterial wilt disease in about 200 plant species (1), including, but not limited to, tomato, potato, banana, peanut, pepper, and eggplant. *R. solanacearum* inhabits subtropical, tropical, and temperate regions and produces four phylogenetically distinct groups that are directly related to their geographical origin. Phylotype I is composed of strains from Asia. The strains of phylotype II are from the Americas. Phylotype II has two clearly recognizable branches, phylotype IIA and phylotype IIB. The members of phylotype III originate from Africa. Phylotype IV isolates are from the Philippines, Japan, Indonesia, and Australia (2–4).

In June 2010, bacterial wilt disease affecting tomato seedlings was reported for the first time by farmers in West Georgia, causing up to 100% plant loss (5). However, no strain from Georgia and/or the Caucasus region has been whole-genome sequenced before.

Herein, we report the draft genome sequences of three *R. solanacearum* strains isolated from different hosts: Geo_57 was isolated from potato, Geo_96 was isolated from tomato, and Geo_99 was isolated from pepper at the Institute of Phytopathology and Biodiversity of Batumi State University (Batumi, Georgia).

*R. solanacearum* isolates were prepared for whole-genome sequencing at the National Center for Disease Control and Public Health (NCDC) Lugar Center. Genomic DNA was extracted from solid-colony culture using a Qiagen DNA MiniPrep kit (Qiagen, Inc.), and whole-genome shotgun sequencing was performed using the Illumina MiSeq platform, according to the manufacturer’s instructions. Paired-end reads were generated and subsequently adapter/quality trimmed using the CLC Genomics Workbench suite version 8.5.1 (CLC bio). Mapped and *de novo* read assemblies were generated and analyzed using CLC bio and the Geneious Basic software suite (Geneious) (6).

Raw data were assembled into 145 contigs for Geo_57, 129 contigs for Geo_96, and 147 contigs for Geo_99. Contigs were annotated by the Rapid Annotations using Subsystems Technology (RAST) server (5).

Georgian isolates have almost 100% average nucleotide identity (ANI) (6, 7) with each other and with strain UY031 (8), indicating the high similarity of these isolates.

Despite the high similarity of Georgian isolates to strain UY031, we found significant differences between the genomes. The chromosomes of the Georgian isolates are 990 nucleotides (nt) shorter; the missing part is in range from positions 2150237 to 2151226 of the reference chromosome. According to reference annotation, this region contains two hypothetical proteins with lengths of 183 and 501 nt. Another sequence difference is found in the chromosome region between positions 324291 and 339697 (15,407 nt). This region in UY031 contains six genes, while the corresponding region in the chromosomes of Georgian strains is presented by one coding sequence with length of 15,429 nt.

The genomes of *R. solanacearum* strains Geo_57, Geo_96, and Geo_99 are made available for further cross-strain comparisons with other global representatives of *R. solanacearum*.

### Accession number(s).

The whole-genome sequences for three Georgian isolates of *R. solanacearum* have been deposited in the GenBank Whole-Genome Shotgun (WGS) database under accession numbers MXAM00000000 (strain Geo_57), MZNA00000000 (strain Geo_96), and MZNB00000000 (strain Geo_99).
